# Supplementary material for: Epigraph hemagglutinin vaccine induces broad cross-reactive immunity against swine H3 influenza virus
Source: Nat Commun. 2021 Feb 22;12:1203. doi: 10.1038/s41467-021-21508-6 (PMC7900167; doi:10.1038/s41467-021-21508-6)
Supplement: Supplementary file 1 — Supplementary Information [file 41467_2021_21508_MOESM1_ESM.pdf]

Epigraph Hemagglutinin Vaccine Induces Broad Cross-reactive Immunity Against Swine H3 Influenza Virus

Brianna L. Bullard[1], Brigette N. Corder[1], Jennifer DeBeauchamp[2], Adam Rubrum[2], Bette Korber[3], Richard Webby[2], and Eric A. Weaver[1]*

[1]School of Biological Sciences, Nebraska Center for Virology, University of Nebraska, Lincoln, NE, 68503, USA
[2]St. Jude Children's Research Hospital, Memphis, TN, 38105, USA
[3]Los Alamos National Laboratory, Los Alamos, New Mexico, 87545, USA

*Correspondence: eweaver2@unl.edu

Vaccine	vp/ml	IFU/mL	vp:IFU ratio
Epigraph 1	4.8e11	6.0e9	79:1
Epigraph 2	1.2e12	1.3e10	90:1
Epigraph 3	1.5e12	1.3e10	117:1
TX98	3.6e12	5.8e10	62:1

Supp. Table 1. **Infectivity of recombinant Ad viral stocks.** All three epigraph immunogens and the Texas/1998 (TX98) wild type HA comparator were cloned into a replication-defective Adenovirus type 5 (Ad) vector and the virus particles (vp) to infectious unit (IFU) ratio was determined to confirm equivalent stock infectivity.

Virus Strain	% amino acid identity to					
					FluSure Strains	
	Epigraph1	Epigraph2	Epigraph3	TX98	Minnesota /2012	North Carolina/2012
A/swine/Ohio/09SW73E/2009 (IV)	96.6	87.6	84.6	91.3	95.8	95.2
A/swine/Manitoba/00446/2005 (IV)	98.2	88.2	85.3	92.2	97.5	96.8
A/swine/Ohio/11SW87/2011 (IV-A)	98.2	88.2	84.3	89.8	95.1	99.3
A/swine/Wyoming/A01444562/2013 (IV-A)	97.9	88.5	84.5	89.8	95.1	98.9
A/swine/Nebraska/4B-1113-P15/2013 (IV-A)	96.5	87.5	84.1	88.9	93.6	97.5
A/swine/North Carolina/A01270394/2012 (IV-A; FluSure)	98.6	88.7	84.3	90.1	95.4	100.0
A/swine/Minnesota/A01270872/2012 (IV-B; FluSure)	96.8	87.5	85.7	91.0	100.0	95.4
A/swine/Minnesota/A01432544/2013 (IV-B)	95.2	87.6	85.9	89.1	95.2	93.8
A/swine/Indiana/A01202866/2011 (IV-C)	97.0	86.7	85.2	91.0	95.8	95.6
A/swine/Minnesota/3908-2/2011 (IV-E)	96.5	86.7	85.3	90.8	95.1	95.1
A/swine/Kansas/11-110529/2011 (IV-F)	95.6	86.6	84.6	90.8	93.8	94.5
A/swine/Texas/4199-2/1998 (I)	91.5	87.5	84.5	100.0	91.0	90.1
A/swine/North Carolina/35922/1998 (I)	92.6	88.0	84.1	96.6	91.7	91.2
A/Swine/Colorado/23619/1999 (II)	91.5	89.2	86.4	93.0	91.7	90.3
A/swine/Texas/A01785781/2018 (Human-like)	88.2	90.8	86.4	88.9	87.8	86.7
A/Ohio/28/2016 (Human-like)	88.5	91.2	86.9	89.4	88.0	87.1
A/swine/Colorado/1/1977 (Human-like)	88.3	85.7	85.2	90.8	87.6	87.6
A/swine/Denmark/101501-1/2011 (Eurasian)	81.6	80.2	82.3	85.2	81.4	80.7
A/swine/Italy/520/1985 (Eurasian)	85.9	83.7	83.9	88.3	85.3	85.3
A/swine/Hong_Kong/4/1976 (Eurasian)	85.3	83.2	82.5	87.8	84.6	84.8
A/swine/Italy/1385-1/1995 (Eurasian)	85.2	83.4	85.2	86.2	84.1	84.3

Supp. Table 2. Percent identity between the vaccine immunogens and the panel of swH3 representative viruses. The percent identity was determined for each vaccine HA and the swH3 virus strains used the HI assay.

A

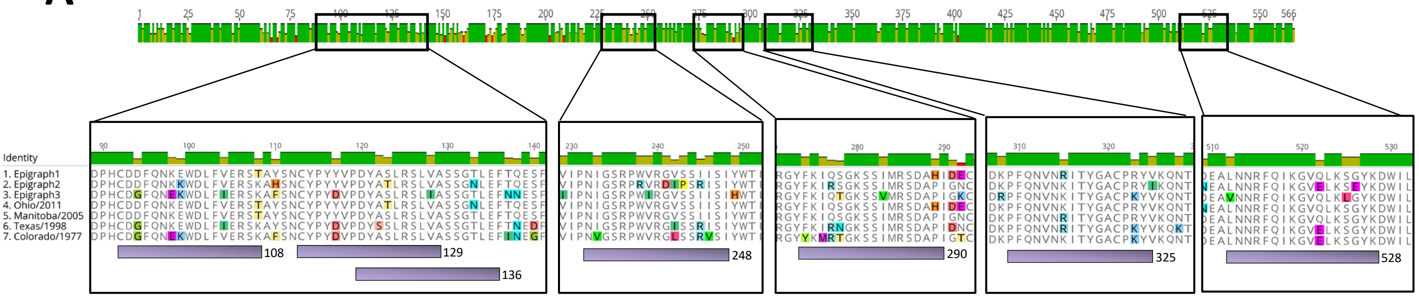

B

Epitope sequence (120-128)	Virus Strain w/ epitope	Percent of protein sequence matches at 100% identity
DYASLRSLV	Epigraph 1	44.01% (687/1561)
	Manitoba/2005	
	Colorado/1977	
DYATLRSLV	Epigraph 2	21.20% (331/1561)
	Ohio/2011	
DYASLRSLI	Epigraph 3	4.16% (65/1561)
DYSSLRSLV	Texas/1998	3.20% (50/1561)

Supp. Fig. 1. T-cell mapping epitope characterization for the swH3 strains. (A) The protein alignment of each positive peptide (>50 spot forming cells per million) and it's relative location on the HA protein is shown. The peptide number designates the position of the last amino acid in the peptide on the total HA protein. (B) The epitope conservancy of each immunodominant epitope 9-mer (amino acid 120-128) in the swH3 sequence population (1561 sequences) was determined using the Immune Epitope Database and Analysis Resource (IEDB).

Virus Strain	% amino acid identity to					
					FluSure Strains	
	Epigraph1	Epigraph2	Epigraph3	TX98	Minnesota /2012	North Carolina/2012
A/Aichi/2/1968	86.7	83.9	84.1	89.0	85.7	86.0
A/Texas/1/1977	89.8	86.4	86.2	92.4	89.8	89.0
A/Mississippi/1/1985	91.5	87.6	86.0	94.2	90.8	90.5
A/Sydney/5/1997	92.9	88.9	86.4	94.9	92.9	91.5
A/Nanchang/933/1995	93.6	89.6	85.3	97.0	93.3	92.2
A/Beijing/352/1989	92.0	87.8	84.8	95.9	91.7	90.6
A/Ann_Arbor/3/1993	90.0	86.3	82.4	94.5	89.7	88.8

Supp. Table 3. Percent identity between the vaccine immunogens and the panel of huH3 representative viruses. The percent identity was determined for each vaccine HA and the huH3 virus strains used the HI assay.

A

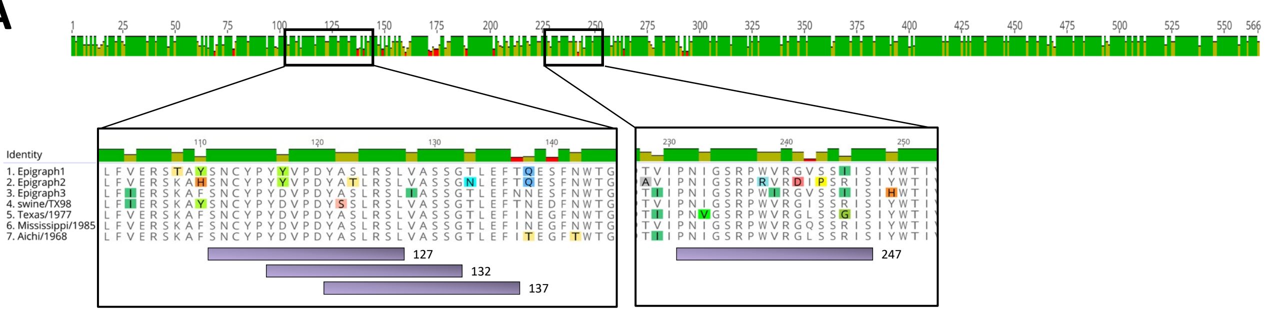

B

Epitope sequence (120-128)	Virus Strain w/ epitope	Percent of protein sequence matches at 100% identity
DYASLRSLV	Epigragh 1	93.68% (5348/5709)
	Texas/1977	
	Mississippi/1985	
	Aichi/1968	
DYATLRSLV	Epigraph 2	0.16% (9/5709)
DYASLRSLI	Epigraph 3	1.68% (96/5709)
DYSSLRSLV	swine/TX98	0.04% (2/5709)

Supp. Fig. 2. T-cell mapping epitope characterization for the human H3 strains. (A) The protein alignment of each positive peptide (>50 spot forming cells per million) and it's relative location on the HA protein is shown. The peptide number designates the position of the last amino acid in the peptide on the total HA protein. (B) The epitope conservancy of each immunodominant epitope 9-mer (amino acid 120-128) in the huH3 sequence population (5709 sequences) was determined using the Immune Epitope Database and Analysis Resource (IEDB).

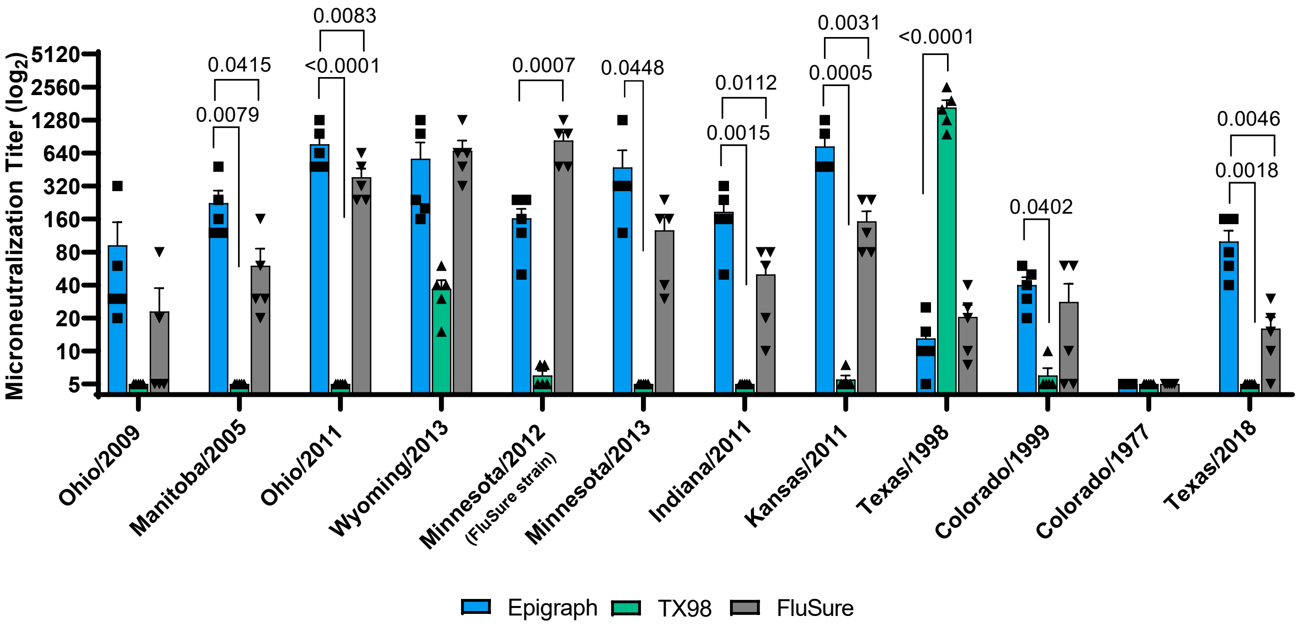

Supp. Fig. 3. Microneutralization titers after prime boost immunizations in swine. The swine sera after boosting was analyzed for the ability to neutralize swH3 strains using a microneutralization assay (n=5; one-way ANOVA with Tukey multiple comparison compared to the epigraph group). Data are presented as the mean with standard error (SEM).

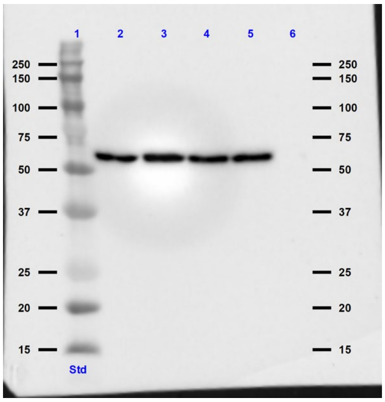

Anti-HA

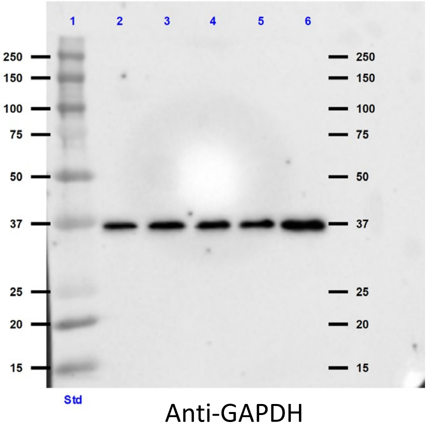

Anti-GAPDH

Supp. Fig. 4: Full western blot images from Figure 1B.
