# Supplementary material for: Epigraph hemagglutinin vaccine induces broad cross-reactive immunity against swine H3 influenza virus
Source: Nat Commun. 2021 Feb 22;12:1203. doi: 10.1038/s41467-021-21508-6 (PMC7900167; doi:10.1038/s41467-021-21508-6)
Supplement: Supplementary file 3 — Reporting summary [file 41467_2021_21508_MOESM3_ESM.pdf]

nature research

Corresponding author(s): Eric A Weaver

Last updated by author(s): Dec 8, 2020

Reporting Summary

Nature Research wishes to improve the reproducibility of the work that we publish. This form provides structure for consistency and transparency in reporting. For further information on Nature Research policies, see our Editorial Policies and the Editorial Policy Checklist.

Statistics

For all statistical analyses, confirm that the following items are present in the figure legend, table legend, main text, or Methods section.

n/a	Confirmed	
☐	☒	The exact sample size (n) for each experimental group/condition, given as a discrete number and unit of measurement
☐	☒	A statement on whether measurements were taken from distinct samples or whether the same sample was measured repeatedly
☐	☒	The statistical test(s) used AND whether they are one- or two-sided<br>*Only common tests should be described solely by name; describe more complex techniques in the Methods section.*
☒	☐	A description of all covariates tested
☒	☐	A description of any assumptions or corrections, such as tests of normality and adjustment for multiple comparisons
☐	☒	A full description of the statistical parameters including central tendency (e.g. means) or other basic estimates (e.g. regression coefficient) AND variation (e.g. standard deviation) or associated estimates of uncertainty (e.g. confidence intervals)
☐	☒	For null hypothesis testing, the test statistic (e.g. F, t, r) with confidence intervals, effect sizes, degrees of freedom and P value noted<br>*Give P values as exact values whenever suitable.*
☒	☐	For Bayesian analysis, information on the choice of priors and Markov chain Monte Carlo settings
☒	☐	For hierarchical and complex designs, identification of the appropriate level for tests and full reporting of outcomes
☒	☐	Estimates of effect sizes (e.g. Cohen's d, Pearson's r), indicating how they were calculated

Our web collection on statistics for biologists contains articles on many of the points above.

Software and code

Policy information about availability of computer code

Data collection	Open source software used in these studies include: Epigraph Vaccine Designer (Los Alamos National Laboratories) and the Influenza Research Database (https://www.fludb.org/brc/home.spg?decorator=influenza)
Data analysis	Data was analyzed using the following software: Geneious 11.1.5, Prism9 GraphPad, and ClustalW

For manuscripts utilizing custom algorithms or software that are central to the research but not yet described in published literature, software must be made available to editors and reviewers. We strongly encourage code deposition in a community repository (e.g. GitHub). See the Nature Research guidelines for submitting code & software for further information.

Data

Policy information about availability of data

All manuscripts must include a data availability statement. This statement should provide the following information, where applicable:

- Accession codes, unique identifiers, or web links for publicly available datasets
- A list of figures that have associated raw data
- A description of any restrictions on data availability

All sequences used to create the immunogens reported in this manuscript are available through the influenza Research Database as described in the materials and methods. All viruses used in these studies are accurately detailed with current strain nomenclature and the sources are described. All other relevant data will be provided by the corresponding author upon request.

Field-specific reporting

Please select the one below that is the best fit for your research. If you are not sure, read the appropriate sections before making your selection.

- [x] Life sciences
- [ ] Behavioural & social sciences
- [ ] Ecological, evolutionary & environmental sciences

For a reference copy of the document with all sections, see nature.com/documents/nr-reporting-summary-flat.pdf

Life sciences study design

All studies must disclose on these points even when the disclosure is negative.

Sample size	We have found that groups of 5 animals are suitable to reach statistical significance. However, as often as possible experiments represent two separate experiments using 5 animals for a total of 10 per analysis (n = 10).
Data exclusions	No data were censored or excluded from the study.
Replication	With the exception of the Western blot in figure 1, all experiments involved the use of animals. The western blot was confirmed several times successfully using polyclonal antisera. Since all of the HA immunogens contained the conserved H3 HA tag, the final western blot was confirmed using an anti-HA Tag HRP conjugated antibody (NB600-391; Novus Biologicals) as this would eliminate variability of antibody binding that could occur using polyclonal antisera.
Randomization	No randomization was performed for the mouse studies. Groups were assigned as shipped from the commercial vendor. The swine were randomly assigned to groups as described in the methods. No identifiable criteria was used in the group assignment of animals.
Blinding	Since this is a preclinical study and we have staff limitations, it was not possible to blind all aspects of the study. Simply put, we just don't have enough staff to act as two independent groups. However, HI results were blinded and the titers were verified by a second investigator. In addition, results were assayed in two separate labs.

Reporting for specific materials, systems and methods

We require information from authors about some types of materials, experimental systems and methods used in many studies. Here, indicate whether each material, system or method listed is relevant to your study. If you are not sure if a list item applies to your research, read the appropriate section before selecting a response.

Materials & experimental systems

n/a	Involved in the study	
[ ]	[x]	Antibodies
[ ]	[x]	Eukaryotic cell lines
[x]	[ ]	Palaeontology and archaeology
[ ]	[x]	Animals and other organisms
[x]	[ ]	Human research participants
[x]	[ ]	Clinical data
[x]	[ ]	Dual use research of concern

Methods

n/a	Involved in the study	
[x]	[ ]	ChIP-seq
[x]	[ ]	Flow cytometry
[x]	[ ]	MRI-based neuroimaging

Antibodies

Antibodies used	anti-HA Tag HRP conjugated antibody (NB600-391; Novus Biologicals), anti-GAPDH (Santa Cruz Biotechnology #0411) and goat anti-mouse HRP conjugated antibody (Millipore Sigma #AP308P)
Validation	Antibody NB600-391; Novus Biologicals was validated for Western Blot Analysis and details are described at the following website: https://www.novusbio.com/products/ha-tag-antibody_nb600-391#protocols-faqs<br>According to the manufacturers' website, anti-GAPDH (Santa Cruz Biotechnology #0411) is recommended for detection of GAPDH of human origin by Western Blotting. More information can be found at the following website: https://datasheets.scbt.com/sc-47724.pdf<br>goat anti-mouse HRP conjugated antibody (Millipore Sigma #AP308P) was validated for western blot as recommended by the manufacturers' data sheet which can be found at the following website: https://www.emdmillipore.com/US/en/product/Goat-Anti-Mouse-IgG-Antibody-H+L-HRP-conjugate,MM_NF-AP308P#anchor_DS

Eukaryotic cell lines

Policy information about cell lines

Cell line source(s)	293 cells were received from Microbix (265 Watline Avenue Mississauga, ON) and MDCK cells were received from the International Reagent Resource Repository. BJ5183 Electroporation-Competent Cells (Cat. No. 200154) were used for homologous recombination
Authentication	MDCK cells were obtained through the International Reagent Resource repository. Authentication was performed using Species Identification by Multiplex PCR Amplification of Cytochrome C Oxidase Subunit I Gene and mycoplasma testing. More information is available at the following website: https://www.internationalreagentresource.org/ProductInformationSheet/tabid/1678/Default.aspx?doc=72837.pdf<br><br>The 293 cells were deposited to ATCC by FL Graham and were characterized through chromosal analyses. The following was taken from the ATCC website: This is a hypotriploid human cell line. The modal chromosome number was 64, occurring in 30% of cells. The rate of cells with higher ploidies was 4.2 %. The der(1)t(1;15) (q42;q13), der(19)t(3;19) (q12;q13), der(12)t (8;12) (q22;p13), and four other marker chromosomes were common to most cells. Five other markers occurred in some cells only. The marker der(1) and M8 (or Xq+) were often paired. There were four copies of N17 and N22. Noticeably in addition to three copies of X chromosomes, there were paired Xq+, and a single Xp+ in most cells. We received the low passage 293 cells directly from FL Graham, thorough his biotech company AdVec that is no longer in operation and was purchased by Microbix.
Mycoplasma contamination	BJ5183 cells were not tested for mycoplama as this is not routinely performed for bacterial species. However, when preparing electrocompetent cells, a single colony is selected in order to eliminate contamination. 293 cells have not recently been tested for mycolasma, but were only used for virus growth and western blots for protein expression. MDCK cells tested negative for mycoplasma.
Commonly misidentified lines<br>(See ICLAC register)	No commonly misidentified cell lines were used in this study.

Animals and other organisms

Policy information about studies involving animals; ARRIVE guidelines recommended for reporting animal research

Laboratory animals	Female BALB/c mice ages 6-8 weeks were purchased from Jackson Laboratory. Outbred male and female pigs aged 3 weeks were purchased from Audubon Manning Veterinary Clinic (AMVC). The mice are housed in a Tecniplast IVC caging system with recycled paper bedding (Tekfresh) from Envigo and fed a standard rodent chow (diet number 2016) from Envigo. Enrichment items can include a kim wipe, nyla bone, or plastic hut. Temperatures range from 68-72 degrees Fahrenheit and 30-70% humidity. Animals are maintained on a 14 hour light and 10 hour dark cycle.
Wild animals	No wild animals were used in these studies.
Field-collected samples	No field collected samples were used in the study.
Ethics oversight	All biological procedures were reviewed and approved by the Institutional Biosafety Committee (IBC) at the University of Nebraska, Lincoln (Protocol: 619). Mice and swine were housed in the Life Sciences Annex building on the University of Nebraska – Lincoln (UNL) campus under the Association for Assessment and Accreditation of Laboratory Animal Care International (AAALAC) guidelines. The protocols were approved by the UNL Institutional Animal Care and Use Committee (IACUC) (Project ID 1217, 1717, and 1879). All animal experiments were carried out according to the provisions of the Animal Welfare Act, PHS Animal Welfare Policy, the principles of the NIH Guide for the Care and Use of Laboratory Animals, and the policies and procedures of UNL.

Note that full information on the approval of the study protocol must also be provided in the manuscript.
